# Concurrent classical and hypervirulent *Klebsiella pneumoniae* infection in distinct host niches revealed by a rapid nanopore whole-genome and plasmid sequencing method

**DOI:** 10.3389/fcimb.2025.1633833

**Published:** 2025-10-13

**Authors:** Lu Wang, Qiqi Wang, Zizhen Tang, Yuyun Wang, Yuanqing Qu, Danli Wen, Qiulei Zhong, Huan Hu, Yuan Liu, Miao He

**Affiliations:** ^1^ Department of Laboratory Medicine, General Hospital of the Western Theater Command of the People’s Liberation Army (PLA), Chengdu, Sichuan, China; ^2^ Institute of Blood Transfusion, Chinese Academy of Medical Sciences, Chengdu, Sichuan, China; ^3^ Key Laboratory of Bioresources and Ecoenvironment (Ministry of Education), College of Life Sciences, Sichuan University, Chengdu, China; ^4^ Genepore Technologies Co., Ltd., Chengdu, Sichuan, China

**Keywords:** hypervirulent *Klebsiella pneumoniae*, infectious disease, whole-genome nanopore sequencing, endogenous endophthalmitis, within-host strain replacement

## Abstract

**Introduction:**

This study reports a rare case of dual-strain Klebsiella pneumoniae infection involving genetically distinct isolates from the lung and eye of a previously immunocompetent patient.

**Methods:**

A rapid nanopore sequencing workflow on the Gseq-500 platform was used for whole-genome and plasmid sequencing. Comparative genomic and phylogenetic analyses assessed isolate relatedness and identified virulence and resistance determinants.

**Results:**

The lung isolate (KP-L) belonged to the hypervirulent ST23/KL1 lineage and carried a large non-conjugative plasmid encoding rmpA, rmpA2, iuc, iro, and peg-344, together with chromosomal yersiniabactin. In contrast, the eye isolate (KP-E) was a classical ST133/KL116 strain lacking known hypervirulence markers but harboring plasmids encoding heavy-metal resistance genes. Despite the absence of hypervirulence factors, KP-E caused severe endophthalmitis requiring enucleation, underscoring the pathogenic potential of classical strains in immune-privileged sites. Comparative genomic and phylogenetic analyses confirmed that the two isolates were not clonally related.

**Discussion:**

We propose a plausible infection trajectory involving initial hypervirulent K. pneumoniae (hvKp) dissemination followed by niche-specific replacement by Classical K. pneumoniae (cKp) under antibiotic pressure. This case underscores that severe infections can arise from genetically “low-virulence” strains in certain host environments. Comprehensive genomic surveillance and aggressive clinical management strategies remain crucial for improving patient prognosis and understanding pathogen adaptation mechanisms within host niches.

## 1 Introduction

*Klebsiella pneumoniae* encompasses both classical strains (cKp) and hypervirulent strains (hvKp), distinguished by their pathogenic profiles. Classical *K. pneumoniae* typically causes opportunistic hospital-acquired infections (e.g. pneumonia, urinary tract infections) in immunocompromised patients, whereas hvKp can invade healthy hosts (Russo and Marr, 2019) and cause severe community-acquired infections. Despite ongoing research into the differentiation between hvKp and cKp, there remains no universally accepted consensus on their definitive classification (Harada and Doi, 2018). hvKp is frequently associated with a hypermucoviscous phenotype, predominantly exhibits KL1 or KL2 capsular serotypes, and often harbors a range of virulence determinants. However, these phenotypic features alone are insufficient to definitively distinguish hvKp from cKp (Harada and Doi, 2018). Molecularly, hvKp strains are often defined by the presence of specific virulence determinants. Large virulence plasmids—such as pK2044 (Wyres et al., 2020; Russo et al., 2018)from the KL1 strain NTUH-K2044 and pLVPK from the K2 strain CG43—carry genes that collectively confer the hypervirulent phenotype (Russo et al., 2024; Russo and Marr, 2019) . Notably, the presence of the plasmid-borne regulators *rmpA/rmpA2* (which enhance capsule production and hypermucoviscosity), the siderophore systems aerobactin (*iucABCD/iutA*) and salmochelin (*iroBCDN*), and the plasmid gene *peg-344* are highly specific markers of hvKp (Russo et al., 2024). Chromosomally, many hvKp also harbor integrative genomic islands encoding additional virulence factors, such as the yersiniabactin siderophore locus, typically located on an integrative conjugative element (ICE), and sometimes the colibactin genotoxin. These factors further enhance the pathogen’s ability to obtain nutrients such as iron and cause tissue injury. In contrast, cKp strains generally lack these virulence determinants, display diverse capsular types, and rarely cause metastatic infections (Nguyen et al., 2024).

Clinically, hvKp is renowned for causing pyogenic liver abscesses (PLA) that can metastasize to other sites, leading to complications such as endogenous endophthalmitis and meningitis. Endogenous endophthalmitis due to hvKp has a devastating prognosis – in patients with liver abscess-associated endophthalmitis, up to ~41% of eyes require evisceration or enucleation despite antibiotic therapy (Yang et al., 2007). This poor outcome is attributed to the aggressive nature of hvKp infection combined with the eye being an immune-privileged site, where limited immune surveillance can allow unchecked bacterial growth and inflammation (Forrester and Xu, 2012; Streilein, 2003). However, the pathogenic mechanisms in such immune-protected sites such as the eye or brain remain poorly understood, and there is a critical lack of systematic studies comparing strain-specific virulence in these niches.

In this context, we describe a rare case of concurrent lung and eye infections in the same patient caused by two genetically distinct *K. pneumoniae* strains. Whole-genome sequencing (WGS) revealed entirely different genetic backgrounds: the lung isolate belonged to a hypervirulent ST23/KL1 lineage, whereas the eye isolate was a classical ST133/KL116 strain. Our aim was to compare these isolates and delineate differences in their virulence factors, pathogenic potential, and adaptation to their respective niches. By elucidating these differences, we seek to understand how hvKp and cKp behave and evolve during infection in immune-privileged sites, thereby addressing the current gap in knowledge of hvKp pathogenesis.

## 2 Materials and methods

### 2.1 Patient and clinical course

**Figure 1:**
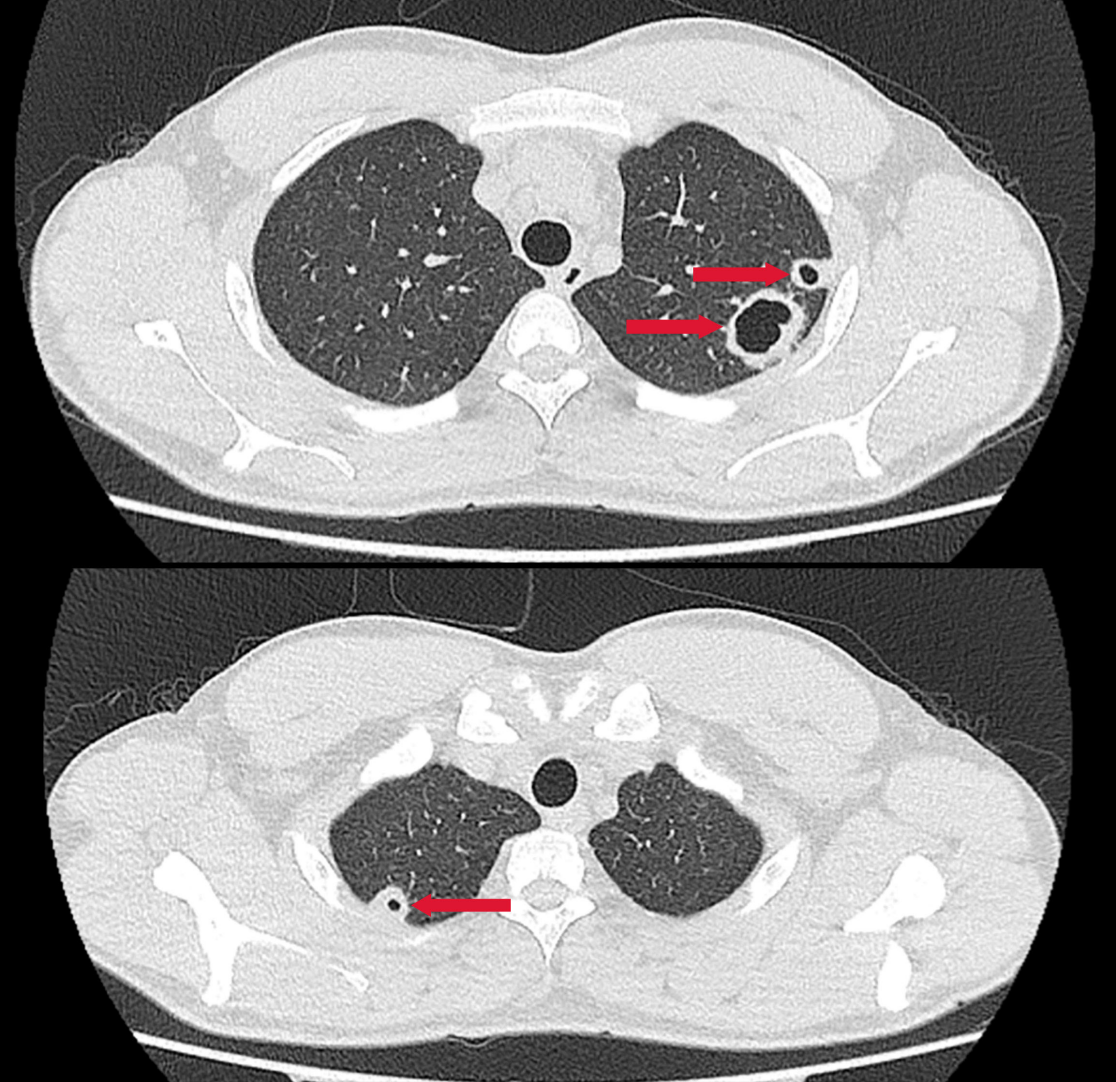
Chest CT showing multiple nodular and patchy high-density opacities in both lungs with ill-defined margins. Several lesions exhibit cavitation. Red arrows indicate the lesion areas.

**Figure 2:**
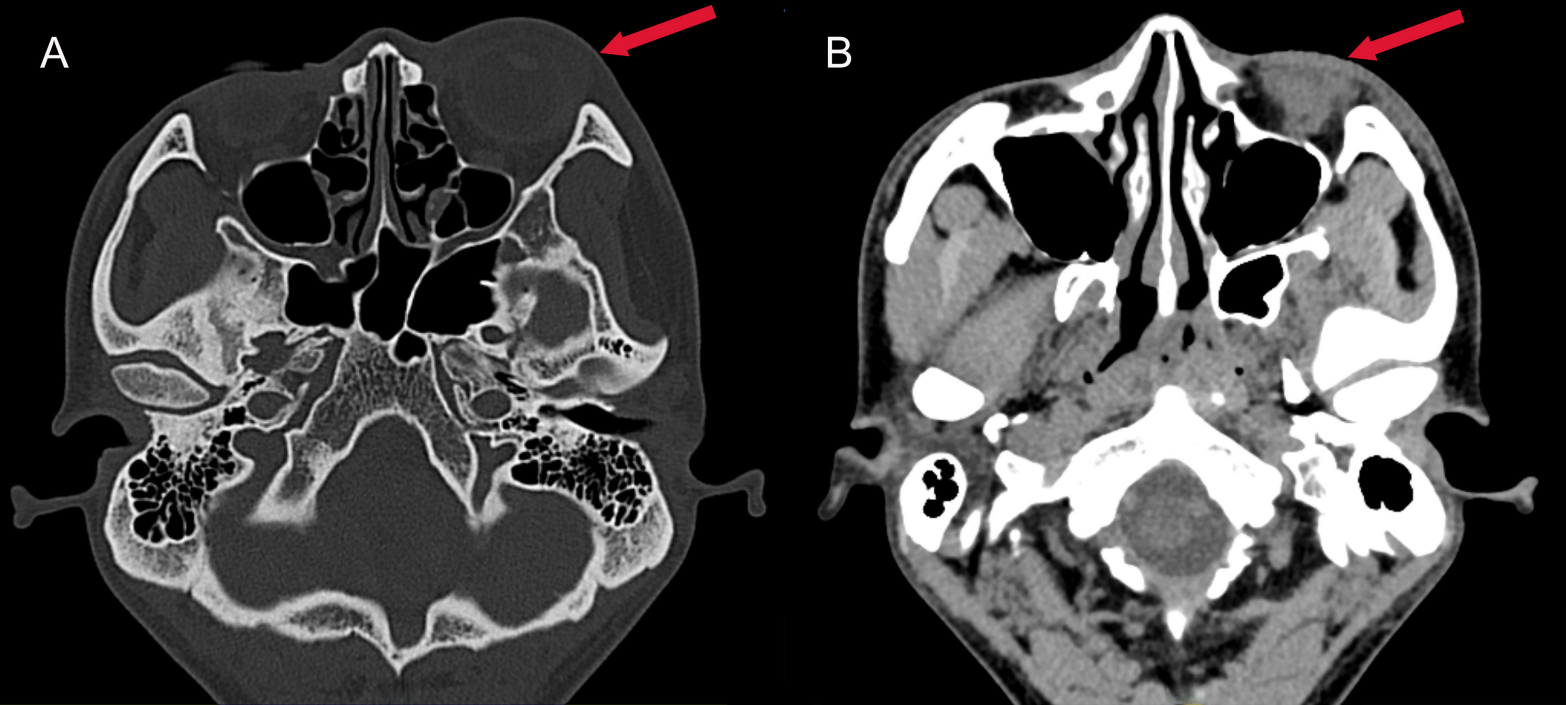
Axial CT images of the head. **(A)** Bone window CT revealing swelling of the left eye (red arrow); **(B)** Contrast-enhanced soft tissue window showing exudation in the left eye (red arrow), characterized by a hypodense area in the periorbital soft tissues.

**Figure 3:**
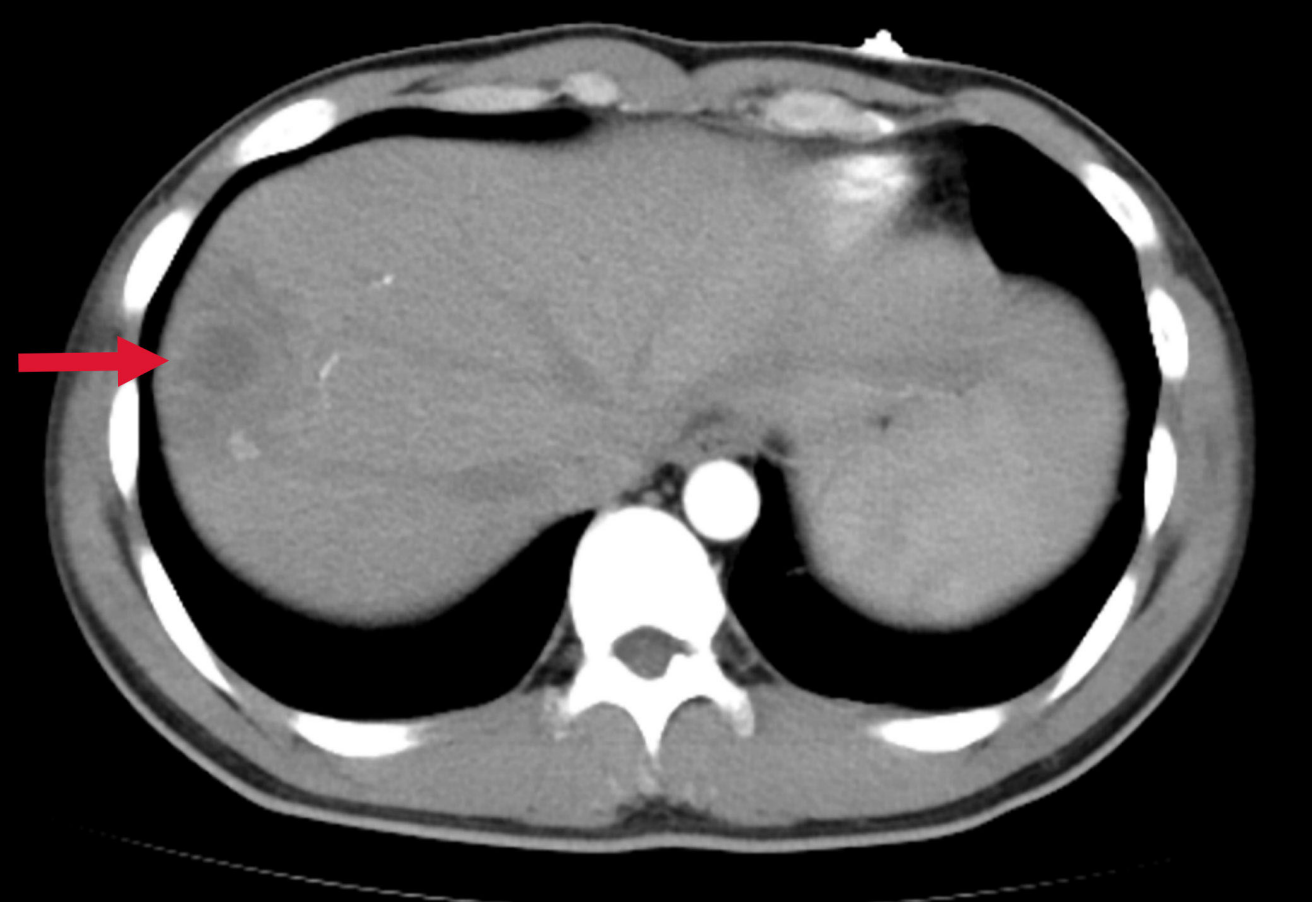
Abdominal CT showing a low-density lesion with ill-defined borders in the right lobe of the liver, suggestive of a hepatic abscess. The red arrow indicates the abscess site.

**Figure 4:**
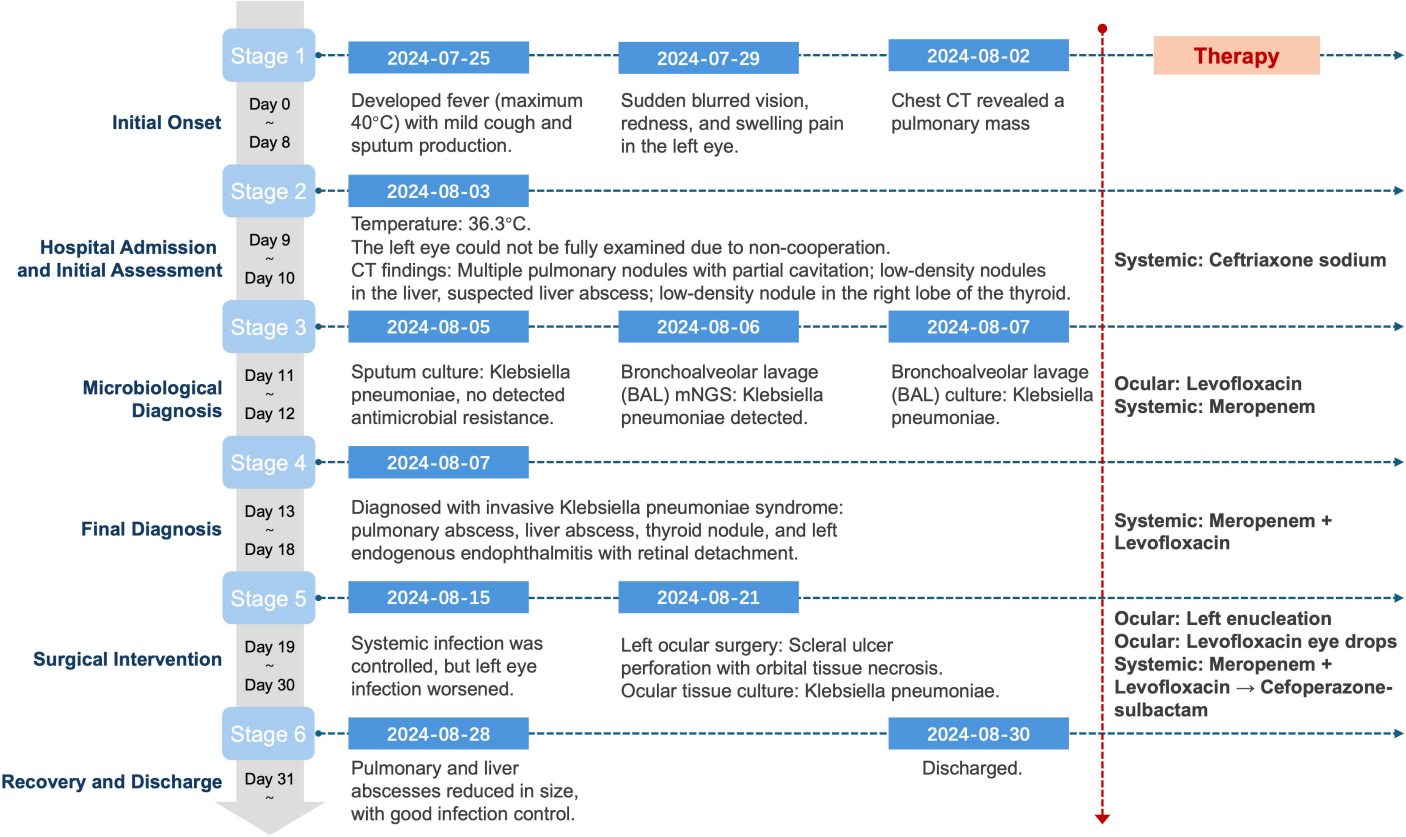
Clinical timeline of disease progression, diagnostic milestones, and antimicrobial interventions in a patient with mixed hypervirulent and classical *K. pneumoniae* infection.

A previously healthy 25-year-old male developed high fever (maximum 40°C) without an identifiable cause while traveling in Jiangxi, China. The fever was accompanied by mild cough and sputum production. Despite self-medicating with over-the-counter cold medication, his symptoms persisted. Five days later, he experienced sudden onset of blurred vision, redness, and painful swelling in his left eye. After nearly a week of persistent symptoms without improvement, he underwent a chest CT scan, which revealed a pulmonary mass, prompting further evaluation. Upon hospital admission on August 2, 2024, CT imaging demonstrated multiple pulmonary nodules with partial cavitation, suggesting a possible infectious etiology (**Figure 1**). Orbital CT revealed an intraocular mass in the left eye, with irregular margins of the left globe and heterogeneous attenuation in the peribulbar and orbital apex tissues (**Figure 2**). Additionally, a low-density hepatic lesion was identified (**Figure 3**), raising suspicion of a liver abscess, and a low-density nodule was detected in the right thyroid lobe. Laboratory tests revealed leukocytosis and significantly elevated inflammatory markers, leading to the initiation of empiric antibiotic therapy with ceftriaxone sodium. Two sets of peripheral blood cultures collected on August 4, 2024 (left and right upper limbs) were negative; blood cultures were not repeated. Due to patient non-cooperation, a complete ophthalmologic evaluation of the left eye was not feasible. Hemodynamics remained stable without vasopressors (SBP 110–130 mmHg/DBP 70–85 mmHg); lactate was 1.2 mmol/L, urine output was ≥1 mL/kg/h, and mental status was normal, indicating no clinical evidence of septic shock. On August 5, 2024, bronchoalveolar lavage fluid (BALF) culture grew *K. pneumoniae* without detected antimicrobial resistance. Metagenomic next-generation sequencing (mNGS) of bronchoalveolar lavage (BAL) fluid further confirmed the presence of *K. pneumoniae.* Given the severity of the infection, the antimicrobial regimen was escalated to meropenem. Despite intensive antimicrobial therapy, the infection progressed rapidly, affecting multiple organ systems. The patient was diagnosed with invasive *K. pneumoniae* syndrome, characterized by pulmonary abscess, liver abscess, thyroid nodule, and endogenous endophthalmitis with retinal detachment. Given the disseminated nature of the infection, the antimicrobial regimen was further adjusted to meropenem plus levofloxacin. Although systemic infection was gradually controlled, the left eye infection continued to deteriorate. The patient developed worsening ocular pain and progressive vision loss. Ophthalmologic evaluation revealed scleral ulcer perforation with extensive orbital tissue necrosis, resulting in irreversible ocular damage. Due to the severity of the infection, on August 21, 2024, the patient underwent left ocular enucleation. Postoperative ocular tissue cultures confirmed *K. pneumoniae* infection. Following surgery, the antimicrobial regimen was adjusted from meropenem plus levofloxacin to cefoperazone-sulbactam. By the time of discharge, follow-up imaging demonstrated a reduction in pulmonary and hepatic abscess size, with adequate infection control. **Figure 4** illustrates the detailed course of disease progression.

### 2.2 Bacterial isolation and culture

Bacterial isolation and species identification: Clinical isolates of *K. pneumoniae* were obtained from the patient’s BALF (representing the lung infection) and from infected tissue collected during left eye enucleation (representing the endophthalmitis). BALF (5 mL) was centrifuged at 3,000 × g for 10 min at 4°C and the pellet resuspended in 0.5 mL sterile normal saline; ocular tissue (~100 mg) was aseptically minced, homogenized in 1 mL sterile normal saline, then centrifuged at 2,000 ×g for 5 min and the supernatant used for plating. Processed material from both specimens was inoculated onto Columbia blood agar and MacConkey agar (Chongqing Ponton Medical Devices Co., Ltd., Chongqing, China) and incubated at 37°C in 5% CO_2_ for 24–48 h with inspections at 24 h and 48 h. Colonies with morphology typical of *K. pneumoniae* (round, convex, smooth to mucoid, 2–3 mm at 24 h); Presumptive colonies from Columbia blood agar and MacConkey agar were Gram-stained (Gram-negative rods) and identified using an automated system (VITEK 2 Compact, GN identification card; bioMérieux, Marcy-l’Étoile, France), which called *K. pneumoniae* for both isolates. These isolates were designated lung isolate (KP-L) and eye isolate (KP-E). Antimicrobial susceptibility testing(AST) was performed by broth microdilution and interpreted according to CLSI M100 (2024) breakpoints (per-drug MICs and interpretations in **Table 1**). Pure cultures were stored at −80°C in glycerol stocks until DNA extraction and downstream analyses.

**Table 1:** Antimicrobial susceptibility (broth microdilution) of KP-L and KP-E with CLSI M100 (2024) interpretations (MICs, mg/L).

Antimicrobial agent	KP-L		KP-E		MIC breakpoint range		
	MIC	interp.	MIC	interp.			
Amoxicillin–clavulanate	≤2	S	8	S	S ≤8/4	I =16/8	R ≥32/16
Piperacillin–tazobactam	≤4	S	≤4	S	S ≤8/4	I =16/4	R ≥32/4
cefuroxime	4	S	8	S	S ≤8	I =16	R ≥32
Cefoxitin	≤4	S	8	S	S ≤8	I =16	R ≥32
Ceftazidime	0.25	S	2	S	S ≤4	I =8	R ≥16
Ceftriaxone	≤0.25	S	0.5	S	S ≤1	I =2	R ≥4
Cefoperazone–sulbactam	≤8	S	≤8	S	S ≤16	I =32	R ≥64
Cefepime	≤0.12	S	≤0.12	S	S ≤2	SDD 4-8	R ≥16
Ertapenem	≤0.12	S	≤0.12	S	S ≤0.5	I =1	R ≥2
Imipenem	1	S	≤0.25	S	S ≤1	I =2	R ≥4
Amikacin	≤2	S	≤2	S	S ≤4	I =8	R ≥16
Levofloxacin	≤0.12	S	≤0.12	S	S ≤0.5	I =1	R ≥2
Tigecycline	1	S	1	S	S ≤2	I =4	R ≥8
Trimethoprim–sulfamethoxazole	≤20	S	≤20	S	S ≤40		R ≥80

MIC, minimum inhibitory concentration; S, susceptible; I, intermediate; R, resistant; SDD, susceptible-dose dependent (cefepime).

Phenotypic characterization and string test: The hypermucoviscosity (“string”) assay was performed according to previously published procedures (Russo and Marr, 2019; Shon et al., 2013): after 24 h of growth on MacConkey agar, a sterile inoculating loop was gently touched to a morphologically typical single colony and then lifted vertically at a constant pace (~1 cm/s); the filament length extending from colony to loop was measured with a millimeter ruler. A string length ≥ 5 mm was interpreted as positive, whereas < 5 mm (including no filament) was considered negative.

### 2.3 Whole-genome sequencing

Bacterial genomic DNA from ocular culture isolates and bronchoalveolar lavage (BAL) fluid was extracted using the TIANamp Stool DNA Kit (DP328; Tiangen Biotech, Beijing, China) according to the manufacturer’s instructions. Concentration was measured by Qubit™ 4 Fluorometer (Thermo Fisher Scientific, Waltham, MA, USA) and integrity by agarose gel electrophoresis; both preparations showed a predominant fragment range of ~6–8 kb, with 94.8 ng/µL (11.38 µg total) for the eye isolate and 20.6 ng/µL (2.47 µg total) for the BAL isolate. Nanopore libraries were prepared with the Universal Sample Preparation Kit for Nanopore Sequencing (Geneus Technologies, Chengdu, China): linear dsDNA was purified with MB-1 magnetic beads (Geneus Technologies, Chengdu, China), end-repair and adapter ligation (Reagents 1/2 and Adapter A–C) ((Geneus Technologies, Chengdu, China) were performed followed by a second MB-1 cleanup, sequencing complexes were assembled per kit instructions, and a final cleanup with MB-2 beads (Geneus Technologies, Chengdu, China) was conducted. Libraries were normalized by gradient dilution, loaded onto a Gseq-500 (Geneus Technologies, Chengdu, China) (Zhou et al., 2025), and run for 5 h at 26°C per the vendor’s recommendations.

### 2.4 Genome assembly

The sequencing data was processed following a multi-step assembly and polishing workflow. Low-quality reads were removed with Prowler (Lee et al., 2021) using -m S -q 12 -g F2. Reads with mean Phred Q < 12 (≈94% per-base accuracy) were excluded; if a read contained one contiguous low-Q (<12) segment, Prowler trimmed that block and returned at most two high-Q fragments, otherwise the read was discarded. All downstream steps used the post-filtering datasets. The filtered reads were initially assembled using Flye v2.9.6 (Kolmogorov et al., 2019) to generate preliminary contigs. NextDenovo v2.5.2 (Hu et al., 2024) was then employed for error correction of the preprocessed reads. To enhance assembly accuracy, minimap2 v2.29 (Li, 2018) was used to align the NextDenovo-corrected reads against the Flye-assembled contigs. Based on the alignment results, raw reads that were either unaligned or mapped near the contig ends were extracted for further processing. These selected reads were subsequently assembled using MIRA v4.0.2. The contigs generated from Flye (Step 2) and MIRA (Step 6) were combined and reassembled using Flye with the –subassemblies option. Finally, the polished assembly underwent nextPolish v1.4.1 (Hu et al., 2020) refinement to further improve sequencing accuracy.

### 2.5 Genomic features and phylogenetic characterization

Functional annotation of the assembled genomes was performed using MicrobeAnnotator v2.0.5 (Ruiz-Perez et al., 2021). Subsequent analyses focused on the systematic identification of virulence and antimicrobial resistance genes. Virulence factors were identified by aligning the assemblies to the Virulence Factor Database (VFDB) (Liu et al., 2019) using Abricate v1.0.1, with particular emphasis on key virulence determinants associated with *K. pneumoniae*. Antimicrobial resistance (AMR) genes were comprehensively characterized using a combination of AMRFinderPlus v4.0.19 (Feldgarden et al., 2019) (NCBI database, April 16, 2024 release) and Abricate v1.0.1 with integrated CARD (Alcock et al., 2020) and MEGARes 2.0 databases (Doster et al., 2020). This dual approach enabled robust detection of β-lactamase genes (e.g., *blaSHV*, *blaKPC*) and other resistance determinants, as well as assessment of metal resistance operons, including the arsenic resistance operon (ars). Sequence types (STs) were determined using multilocus sequence typing (MLST) based on seven housekeeping genes via the MLST 2.0 (Larsen et al., 2012) webserver hosted by the Center for Genomic Epidemiology (https://cge.food.dtu.dk). Capsular polysaccharide (K) locus types were assigned using the Kaptive Web server (https://kaptive-web.erc.monash.edu) (Lam et al., 2022), which matches the *K. pneumoniae* capsular synthesis locus (K-locus) against a curated reference database. The polished assemblies were screened with Kleborate v3.2.4 (Lam et al., 2021) to compute the standardized virulence score (0–5; based on *ybt/clb/iuc*) for each isolate. Phylogenetic relationships among isolates were inferred using the BUSCO_phylogenomics pipeline (Manni et al., 2021). A set of conserved single-copy orthologs from the Bacteria odb10 database was extracted to construct multiple sequence alignments, and a maximum likelihood (ML) phylogenetic tree was generated to resolve the evolutionary relationships.

## 3 Results

### 3.1 Nanopore sequencing output and base-level quality

**Figure 5:**
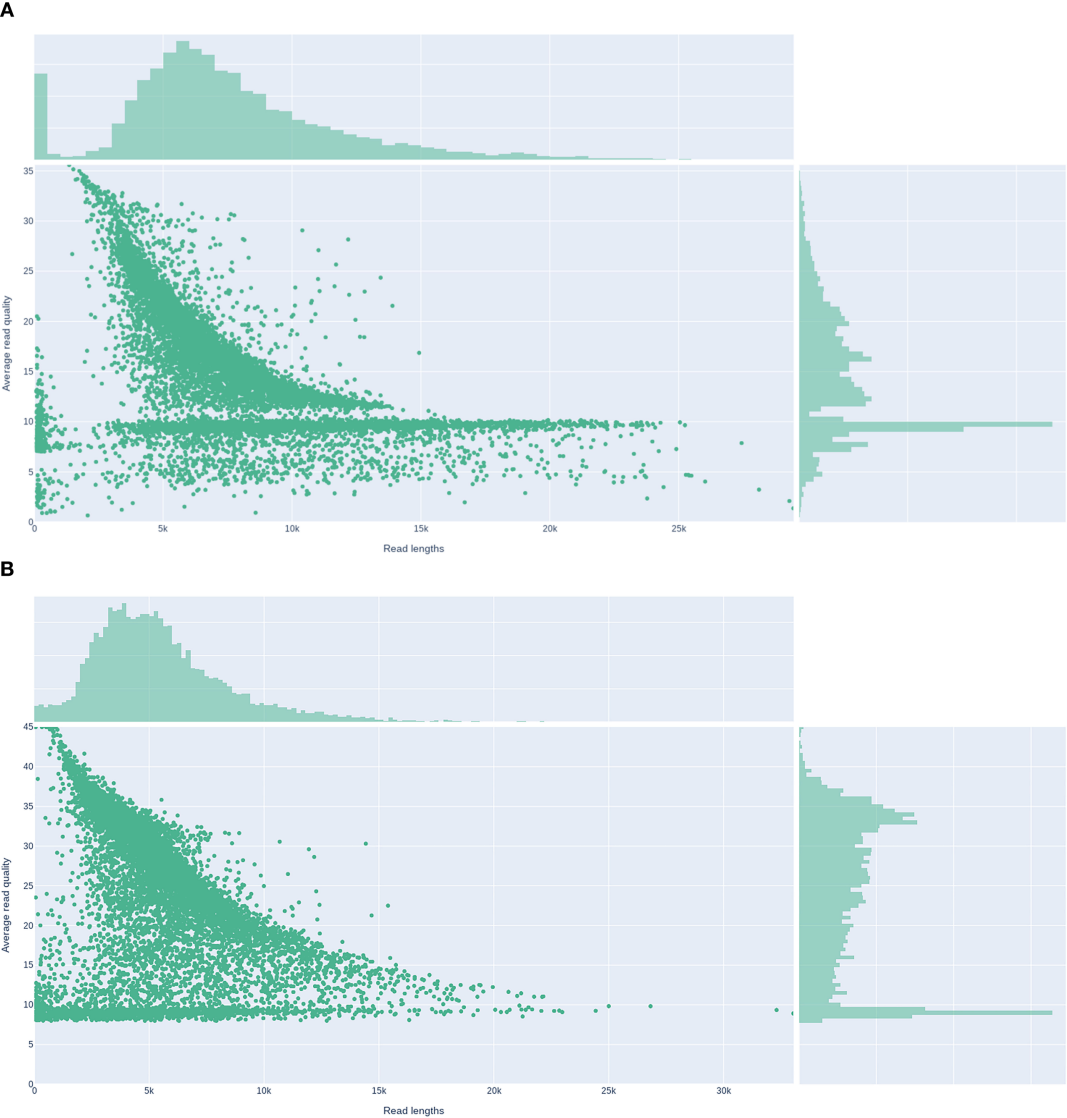
Read-length versus average read-quality distributions for Nanopore datasets. **(A)** Lung isolate KP-L. **(B)** ocular isolate KP-E.

The two isolates were processed with the Nanopore Rapid Barcoding workflow, enabling the entire wet-lab cycle, from DNA extraction to flow-cell loading, to be completed within 24 h. After Prowler filtering (Q ≥ 12), the final datasets comprised 82,485 reads (459.9 Mbp; mean 7.0 kb) for KP-L and 123,909 reads (683.4 Mbp; mean 5.5 kb) for KP-E, corresponding to ~84× and ~130× coverage, respectively. Read-length distributions and base-level quality metrics are shown in **Figure 5** (KP-L: N50 8.7 kb, max 50.9 kb; KP-E: N50 6.5 kb, max 37.6 kb). These long-read datasets were sufficient to generate gap-free assemblies of all replicons, including both the chromosome and plasmids, in a single sequencing run.

### 3.2 Chromosome and plasmid content

Whole−genome assembly showed a marked structural divergence between the isolates. KP−L comprised a single 5.47 Mb chromosome plus one large ~229 kb plasmid, whereas KP−E carried a smaller 5.25 Mb chromosome and two plasmids (~145 kb and ~11 kb). Consequently, the total genome of KP−E was ~220 kb shorter than that of KP−L. Notably, KP−L retained a canonical hvKp−type megaplasmid, while KP−E lacked this element altogether and instead harbored a smaller IncFIB/IncFIIK plasmid of unknown function (**Table 2**).

**Table 2:** Genome assembly statistics for lung (KP−L) and eye (KP−E) isolates.

Isolate	Replicon	Size (bp)	GC content (%)	Cumulative Genome Size (bp)
KP-L (lung)	Chromosome	5,471,644	57.5	5,700,617
	Plasmid pLung	228,973	50.1	
KP-E (eye)	Chromosome	5,248,703	57.6	5,405,113
	Plasmid pEye-1	145,217	52.5	
	Plasmid pEye-2	11,193	55.8	

### 3.3 Genomic relatedness and sequence typing

WGS revealed that the lung and eye infections were caused by two different clones of *K. pneumoniae*. KP-L was identified as ST23 with capsule type KL1 (serotype K1), a canonical hypervirulent lineage (Nguyen et al., 2024). In contrast, KP-E was ST133 with capsule KL116, an unrelated lineage that is generally considered a classical *K. pneumoniae* strain (Mills et al., 2024). Assignment to different sequence types (ST23 vs ST133), which differ at five of the seven MLST loci, indicates that the isolates derive from different lineages (**Table 3**). KP-L achieved a virulence score of 5/5 (consistent with *ybt + clb + iuc*), whereas KP-E scored 0/5 (none detected), quantitatively supporting the hvKp vs cKp assignments (**Supplementary Material**). Phylogenetic analysis confirmed that KP-L and KP-E are genetically distant. This shows the lung and eye isolates did not descend from a common recent ancestor within the host.

**Table 3:** Comparison of MLST alleles between lung (KP-L) and eye (KP-E) isolates.

Locus	Allele(KP-L)	Allele(KP-E)
gapA	gapA_12	gapA_2
infB	infB_1	infB_1
mdh	mdh_1	mdh_1
pgi	pgi_2	pgi_1
phoE	phoE_5	phoE_9
rpoB	rpoB_1	rpoB_4
tonB	tonB_36	tonB_12

### 3.4 Comparative virulence gene profiles

**Figure 6:**
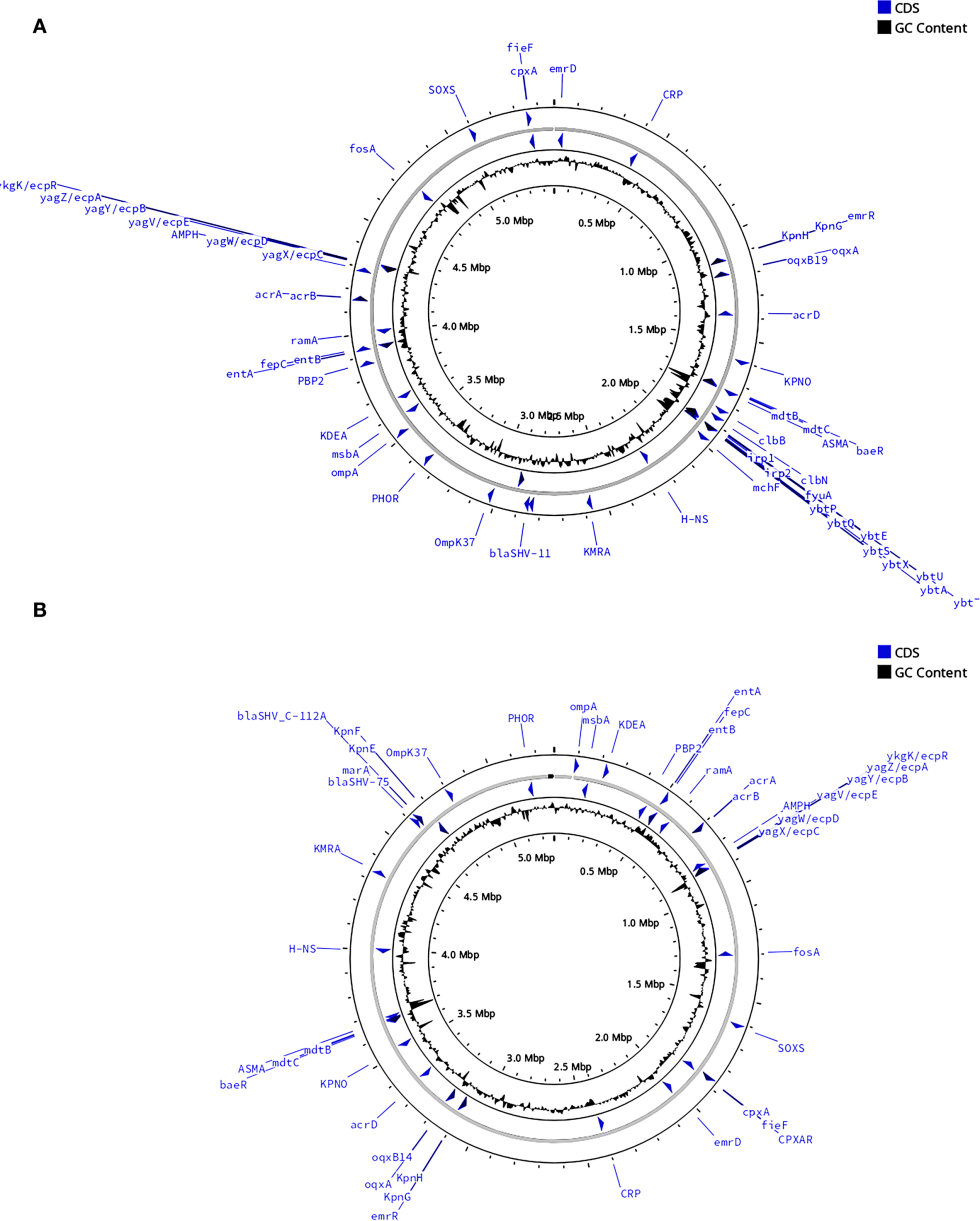
Comparative circular maps of the chromosomal backbones of the lung (KP-L) and eye (KP-E) *K pneumoniae* isolates. **(A)** Chromosome of the ST23-hvKp lung isolate (KP-L). **(B)** Chromosome of the ST133-cKp eye isolate (KP-E). Notable differences between the two isolates are emphasized: KP−L harbors the yersiniabactin (ybt) siderophore island, whereas KP−E carries no yersiniabactin locus. For each map (moving from the outside to the center):1.Outer grey ring—chromosomal scale (tick marks every 100 kb; Mb labels every 0.5 Mb); 2.Blue arrows—coding sequences (CDS) of interest automatically binned by functional class; labels highlight key loci (antimicrobial-resistance determinants, virulence regulators, stress–response genes); 3.Inner grey ring—GC-skew; 4.Innermost black plot—deviation of GC content from the genomic mean; peaks denote atypical, horizontally-acquired islands.

Whole−genome analysis revealed stark contrasts between the two isolates (**Table 4**). KP-L carried a canonical hvKp virulence plasmid, and its chromosome harbored a yersiniabactin (ybt) island, consistent with a hypervirulent genotype. In contrast, KP-E lacked plasmid-borne virulence loci and showed only partial coverage of the ybt locus, yielding a low Kleborate score typical of classical *K. pneumoniae*. Both isolates did possess the *entB* gene for enterobactin (a ubiquitous siderophore in Kp) and chromosomal genes for capsule polysaccharide synthesis (though of different K-types). KP-L harbored the colibactin (*clb*) cluster, whereas KP-E lacked *clb*. Both genomes carried only the intrinsic *blaSHV* β-lactamase with no acquired resistance genes, consistent with their *in vitro* susceptibility to cephalosporins and carbapenems. The overall genomic architecture and annotated virulence/AMR islands are illustrated in **Figure 6**, which highlights the presence of a yersiniabactin island in KP−L (**Figure 6A**) and its absence in KP−E (**Figure 6B**).

**Table 4:** Key virulence-associated loci in the lung vs. eye *K. pneumoniae* isolates.

Virulence factor locus	KP-L (Lung, ST23/KL1)	KP-E (Eye, ST133/KL116)
*rmpA/rmpA2* (capsule regulators, plasmid)	both present	both absent
Aerobactin siderophore (*iucABCD, iutA*, plasmid)	complete cluster	absent
Salmochelin siderophore (*iroBCDN*, plasmid)	complete cluster	absent
*peg-344* (plasmid virulence gene)	present	absent
Yersiniabactin (*irp, ybt, fyuA, chromosomal ICE*)	present	absent
Colibactin (*clb* genotoxin, chromosomal ICE)	present	absent
Capsule type (K antigen)	KL1	KL116
Intrinsic SHV β-lactamase	Yes	Yes
Acquired resistance genes	None detected	None detected

### 3.5 Plasmid architecture and gene maps

**FIGURE 7:**
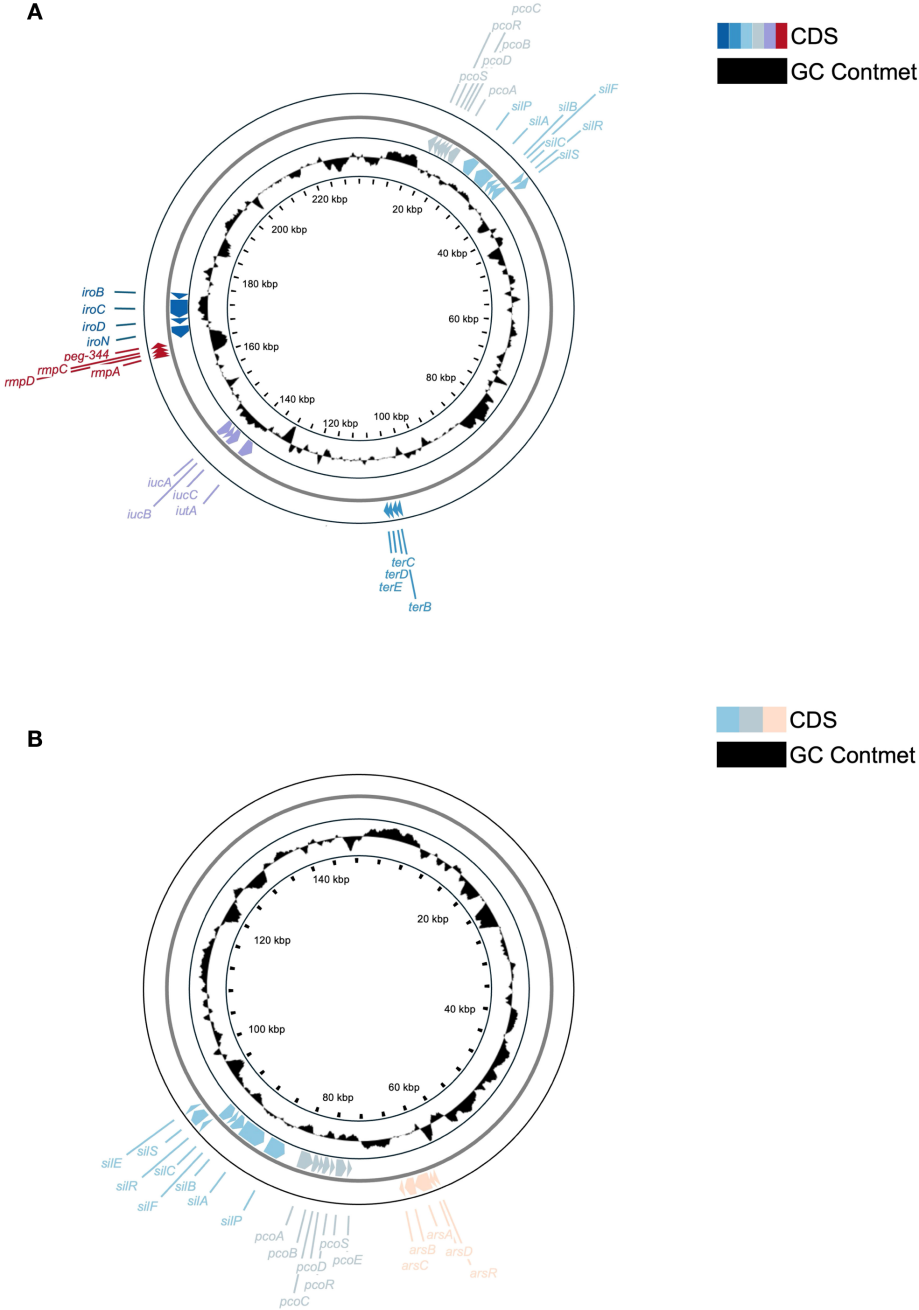
Comparative circular maps of plasmids from the lung (KP-L) and eye (KP-E) (*K*) *pneumoniae* isolates. **(A)** Circular map of the ~229 kb virulence plasmid (pLung) from the ST23/KL1 lung isolate. Rings show coding sequences (colorful) and GC content (black). Key loci are labeled: the aerobactin cluster (*iucA/iucB/iucC/iutA*), salmochelin cluster (*iroB/iroC/iroD/iroN*), mucoid regulators (*rmpA, rmpC; an rmpA2* gene was also present but is not separately labeled), virulence marker *peg-344*, and heavy metal resistance operons for tellurite (*ter*), copper (*pco*), and silver (*sil*). This large IncFIB/IncHI1B plasmid carries the essential hvKp virulence determinants but lacks a complete conjugation module, consistent with known non-transmissible hvKp plasmids. **(B)** Circular map of the ~145 kb plasmid (pEye) from the ST133/KL116 eye isolate. Rings show coding sequences (colorful) and GC content (black). Labeled features include the silver resistance operon (*silABCPRS, silEF*), copper resistance operon (*pcoABCDERS*), and arsenic resistance operon (*arsRDABC*). The presence of these operons indicates an environmental adaptation role. The lack of hvKp loci on pEye corroborates that the eye isolate did not inherit the virulence plasmid carried by the lung isolate.

Consistent with the above, plasmid architectures also differed (**Figure 7**). The ~229 kb plasmid in KP-L plasmid (pLung) is an IncFIB/IncHI1B-type plasmid typical of hvKp (similar to pLVPK). The KP-L plasmid has a pLVPK-like IncHI1B/IncFIB backbone and carries the canonical hvKp virulence cargo—*rmpA/rmpA2, iucABCD–iutA* (aerobactin), *iroBCDN* (salmochelin), and *peg-344* (Lam et al., 2021). The plasmid lacks a complete *tra* region; neither a relaxase (*MOB* family) nor an *oriT* motif was detected, so it is predicted to be non-self-transmissible, consistent with hvKp virulence plasmids that are frequently non-conjugative. Conjugation was not assessed (Lam et al., 2021). Compared with pLVPK (~219 kb), pLung is larger (~229 kb) and displays a duplicated *iuc* cluster, additional toxin–antitoxin systems (e.g., ccdAB), and expanded metal-tolerance islands (e.g., *ter, pco/sil*). **Figure 7A** shows the annotated pLung map. A locus-by-locus comparison with pLVPK is provided in **Supplementary Material**.

In contrast, the ~145 kb plasmid in KP-E (pEye) had an entirely different gene content (**Figure 7B**). It belongs to the IncFIB(K) group (Klebsiella-associated IncFIB) with an IncFIIK backbone – a plasmid type frequently found in classical *K. pneumoniae* (Bi et al., 2018; Leavitt et al., 2010). pEye did not contain *rmpA, iuc, iro, or peg-344*. Instead, pEye encoded a number of genes related to heavy metal resistance and conjugation. Notably, pEye harbors the arsenic resistance operon (arsRADC) in addition to the pco and *sil* operons (the latter two typically co-located on plasmids as the Copper Homeostasis and Silver Resistance Island, CHASRI), features that are associated with improved survival in metal-exposed healthcare settings (e.g., silver-coated devices or copper-treated surfaces) (Finley et al., 2015; Pietsch et al., 2020). pEye also carries several *tra* gene fragments (type IV secretion system components), indicating it was once a conjugative plasmid or can be mobilized by a helper plasmid. However, the *tra* genes on pEye appear incomplete, so autonomous transfer is likely impaired. The small 11 kb plasmid in KP-E encodes a replication protein but no notable factors (likely a cryptic plasmid).

### 3.6 Antibiotic susceptibility–related genomic resistome

AMRFinderPlus assigned chromosomal *blaSHV-11* to KP-L and chromosomal *blaSHV_C-112A* to KP-E, a lower-scoring secondary hit to *blaSHV-75* was also returned. No acquired β-lactamases (e.g., *blaCTX-M, blaKPC/NDM/OXA-48-like*), plasmid-mediated quinolone resistance genes (*e.g., qnr, aac(6′)-Ib-cr*), 16S rRNA methylases (*e.g., armA, rmtB*), *mcr*, or other acquired AMR determinants were detected on the chromosomes or plasmids.

Hits were called using default thresholds. Phenotypic antimicrobial susceptibility testing was performed by broth microdilution and interpreted per CLSI M100 (2024); genotype–phenotype were concordant (**Table 1**). Plasmid-borne metal-tolerance operons are reported separately (KP-L: *ter, pco/sil*; KP-E: *arsRADC, pco/sil*), as they are distinct from antibiotic resistance (see Section 3.5 and **Figure 7**).

### 3.7 Phenotypic correlates

**Figure 8:**
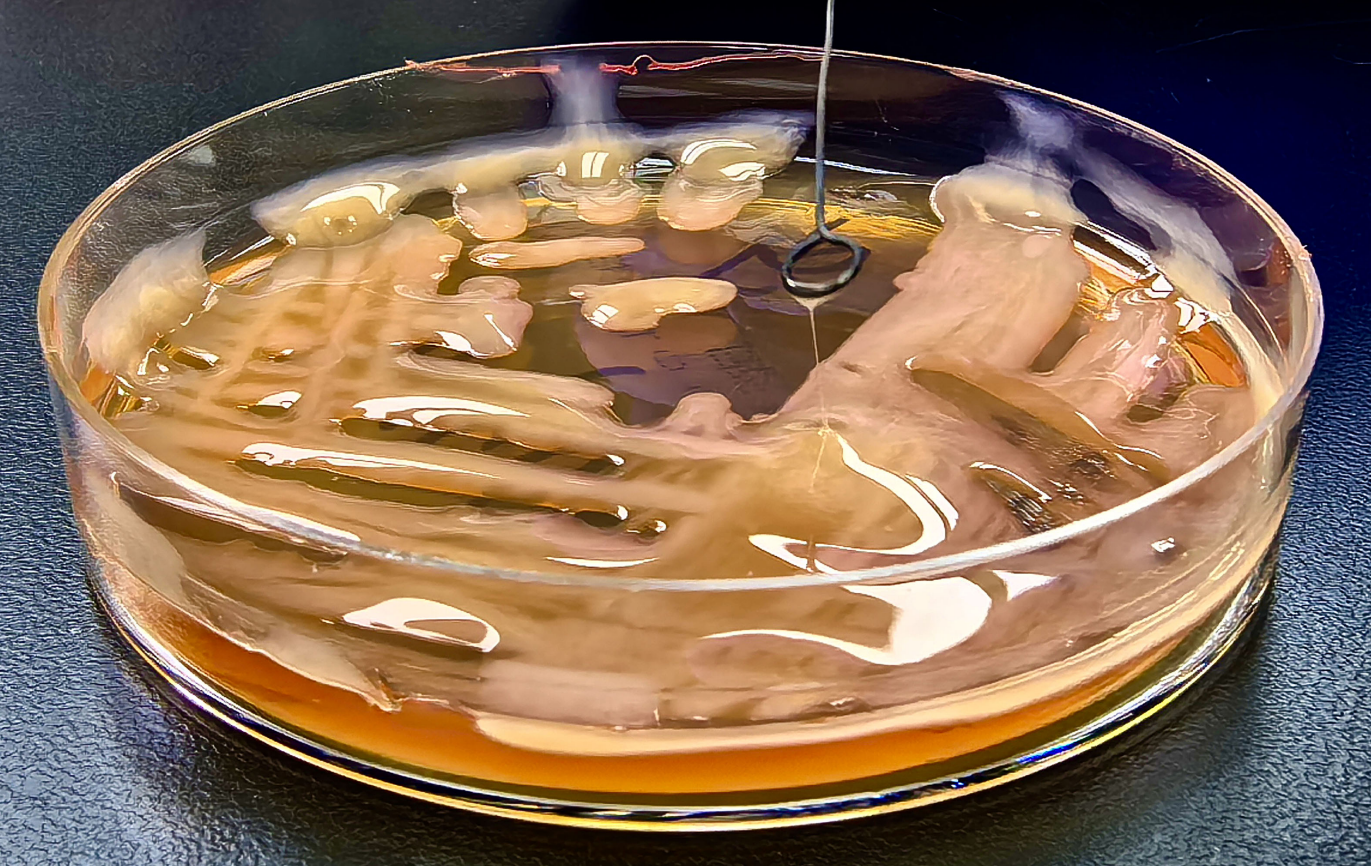
Positive string test of the lung-derived *K. pneumoniae* strain (KP-L) isolated from bronchoalveolar lavage fluid.

The lung strain (KP−L) produced a characteristic hypermucoviscous string measuring ~5 mm (**Figure 8**). In contrast, colonies of the eye isolate (KP−E) yielded a negligible filament and spread as a matte, non−mucoid film on agar. AST by broth microdilution showed susceptibility across the tested panel for both isolates; per-drug MICs and CLSI (2024) interpretations are listed in **Table 1**.

## 4 Discussion

This case highlights an unusual scenario in which a single patient was infected at two sites by *K. pneumoniae* strains with profoundly different virulence characteristics. The clinical course initially resembled a typical hvKp syndrome, with a healthy individual developing pneumonia, metastatic abscesses, and endophthalmitis—features commonly linked to hematogenous spread of a hypervirulent strain (Russo and Marr, 2019). Indeed, the lung isolate was a hypervirulent ST23/KL1 strain, consistent with the predominant hvKp lineage known to cause such invasive disease (ST23 KL1 causes ~82% of hvKp liver abscess cases (Lam et al., 2018)). However, WGS revealed that the endophthalmitis was not caused by the same ST23 clone, but by a genetically distinct ST133 strain lacking the canonical hvKp virulence factors. Endogenous endophthalmitis is one of the first recognized clinical manifestations that distinguishes infections caused by hvKp from those caused by cKp (Liu et al., 1986).Consequently, reports of endogenous endophthalmitis arising from a strain distinct from the hvKp responsible for systemic dissemination are exceedingly rare within the context of hvKp syndrome, rendering this case particularly noteworthy.

### 4.1 Infection dynamics and within-host strain replacement

The mixed-strain finding prompts an analysis of potential infection trajectories. Below, we evaluate three possible pathways for this stepwise polymicrobial infection. Pathway 1: The hvKp (ST23) strain initially caused bacteremia and seeded the eye, but was later replaced by ST133 cKp due to antibiotic pressure and a permissive ocular environment. Pathway 2: Both hvKp and cKp strains entered the bloodstream simultaneously and independently established infections at different anatomical sites. Pathway 3: The lung and eye infections were caused by two unrelated strains, with the ocular ST133 cKp infection introduced directly via exogenous factors. Among these, Pathway 1 best reconciles the observed virulence dynamics—initial aggressive invasion followed by opportunistic takeover—with the patient’s clinical course. The proposed trajectory unfolds as follows:(i) The hvKp (ST23) strain enters the bloodstream and seeds the eye, disrupting the blood–ocular barrier;(ii) Subsequent antibiotic therapy suppresses hvKp, creating an ecological void that is filled by ST133 cKp originating from endogenous flora. Notably, hvKp’s large virulence plasmid imposes a significant metabolic burden (Fan et al., 2022). Such plasmid carriage can incur fitness costs in the absence of antibiotics (San Millan and Maclean, 2017), whereas during therapy, hvKp is more readily suppressed due to its broad susceptibility (Russo and Marr, 2019; Kochan et al., 2023), enabling niche replacement by cKp. Consequently, when broad-spectrum antibiotics are administered, the pansusceptible hvKp strain is rapidly diminished. The damaged ocular environment—characterized by exposed collagen, necrosis, and residual antibiotic concentrations—then becomes a niche where the more resilient ST133 cKp can colonize and proliferate. WGS supports this sequence of events: the lung isolate harbors classical hvKp virulence loci (e.g., *rmpA*, aerobactin) but lacks major resistance genes, while the eye isolate is devoid of virulence loci and instead possesses a broader array of heavy metal resistance genes, suggesting enhanced environmental adaptability.

This two-step mechanism mirrors documented cases of sequential infections, where one pathogen creates a permissive environment for a second pathogen under antibiotic pressure—similar to post-treatment secondary infections. Unfortunately, without strain isolates from blood or other sites at intermediate time points, we cannot pinpoint exactly when or how ST133 was introduced. Blood cultures were obtained after initiation of antibiotics and were not repeated; this may have lowered culture sensitivity and precluded assessment of bacteremia duration, further constraining our ability to time the sequence of events. Notably, the two-step model emphasizes ecological selection rather than higher MICs. Broad-spectrum antibiotics likely reduced the pansusceptible hvKp burden more profoundly, whereas the ocular compartment—with sub-MIC/poorly penetrant drug levels, tissue injury, and biofilm-permissive surfaces—provided a niche in which cKp could persist despite *in vitro* susceptibility. Even when endophthalmitis-related inflammation attenuates immune privilege, the eye retains relatively limited immune effector access, and injury-related environmental changes can still favor colonization. Consistent with this view, both isolates were broadly susceptible on AST (**Table 1**); thus, the observed shift reflects niche-specific fitness/tolerance rather than acquired resistance. In contrast, Pathways 2 and 3 are less consistent with the clinical and genomic findings, and are presented in this report as theoretical alternatives. We consider Pathway 2 unlikely given the genomic distinctiveness and absence of hvKp markers in KP-E. Pathway 3, exogenous direct inoculation, is less plausible due to absence of trauma history and strict sterile ocular conditions pre-enucleation. The host immune privilege likely played a critical role in facilitating KP-E proliferation, as immune-privileged sites offer limited inflammatory surveillance, making even lower-virulence strains highly pathogenic.

Together, these findings suggest a within-host ecological succession, in which antimicrobial pressure and tissue microenvironment collectively reshape pathogen dominance. This observed genomic shift highlights the dynamic interplay between virulence potential and survival fitness, shaped by antimicrobial pressure and the evolving local microenvironment. From a clinical perspective, this case raises critical questions regarding infection trajectory and the optimal timing of microbiological sampling. Notably, the ocular specimen in our case was collected only after systemic antibiotic administration and during surgical intervention, a context that ensured sterility but likely missed an earlier window during which both strains may have coexisted or when the hypervirulent clone was dominant. In bacterial endophthalmitis, previous studies have supported early vitreous or anterior chamber sampling as a means to improve diagnostic sensitivity (Durand, 2017; Jackson et al., 2014), enabling more precise antimicrobial targeting and prompt therapeutic intervention. However, most reports do not define a clear temporal threshold for optimal sampling. This lack of standardized timing can result in missed diagnostic windows and underestimation of strain diversity. Furthermore, invasive sampling carries procedural risks, including potential for secondary complications and, in rare cases, bilateral spread. Despite these concerns, the literature emphasizes that early vitreous sampling, combined with immediate intravitreal antibiotic administration or vitrectomy, can substantially improve clinical outcomes and reduce the need for enucleation (Yoon et al., 2003; Sridhar et al., 2014). Still, this strategy must be carefully considered in patients without overt ocular manifestations, particularly in the early stages of systemic *K. pneumoniae* infection.

To reconcile diagnostic efficacy with patient safety, we propose a tiered diagnostic approach. High-risk individuals—such as those with liver abscesses or severe systemic infection—who exhibit even subtle ocular symptoms should undergo prompt evaluation via minimally invasive anterior chamber sampling. This may permit early microbiological diagnosis while minimizing risk. When endophthalmitis is clinically suspected, intravitreal antibiotics should be administered promptly after diagnostic sampling rather than delayed for culture confirmation. Vitreous sampling should be reserved for cases showing clinical progression or suboptimal response to initial treatment. Implementing this stratified strategy may enable earlier detection of ocular colonization, capture dynamic within-host strain shifts, and facilitate timely precision antimicrobial therapy.

### 4.2 Context-dependent pathogenicity and virulence gene loss

Previous studies have demonstrated that a complete set of hypervirulence plasmid markers is closely associated with severe pathogenicity and high lethality, whereas partial loss of these markers can attenuate virulence (Nassif et al., 1989; Tang et al., 2010). In this case, the eye-derived isolate lacked several key virulence genes, including *rmpA2* and *iucA*, resulting in a virulence genotype more consistent with classical *K. pneumoniae*. However, the clinical outcome—an uncontrolled infection ultimately requiring enucleation—reminds us that virulence is highly context-dependent. The immune-privileged environment of the eye likely played a pivotal role in this outcome. The vitreous chamber, as the interior of the eye, has limited immune cell access and suppressed inflammatory responses in order to preserve vision. While this immune privilege usually protects visual function, it can become a liability during infection (Forrester and Xu, 2012), allowing even moderately virulent bacteria to proliferate with minimal immune interference. In essence, the loss of the hypervirulence plasmid may have rendered the bacterium less virulent in systemic sites such as the bloodstream or lungs. Yet within the eye, this attenuation was insufficient to prevent severe damage. The organism still possessed core pathogenic traits such as a robust K2 capsule, lipopolysaccharide (LPS) endotoxin, and the ability to form biofilms or adhere to surfaces—factors that, within the confined space of the eye, can contribute to abscess formation and tissue necrosis. Moreover, the delayed or eventual inflammatory response can itself cause collateral damage; endophthalmitis is well known for causing vision-threatening inflammation. If the eye isolate had been the only sample available, standard molecular tests might have failed to classify it as hypervirulent, given the absence of *rmpA* and *iucA*, potentially leading clinicians to underestimate its invasive potential. However, the clinical picture—marked by a concurrent liver abscess and disseminated infection—clearly indicated an origin from a hypervirulent *K. pneumoniae* strain. This case highlights a critical point: relying solely on single time-point genetic testing may be misleading. In severe infections with features characteristic of hvKp, such as multifocal abscesses or metastatic spread, clinicians should maintain an aggressive therapeutic approach even if genotypic markers are inconclusive.

### 4.3 Niche adaptation and the fitness trade-off of hypervirulence

To explain the compartmentalized dominance of hvKp and cKp at different anatomical sites within the host, we hypothesize a context-dependent fitness trade-off: bacterial subpopulations lacking virulence genes may gain a selective growth advantage in specific ecological niches during disease progression and under prolonged antimicrobial therapy. Large plasmids can impose a metabolic burden; if the plasmid does not confer a survival advantage under specific conditions, plasmid-free cells might outcompete plasmid-bearing ones (San Millan and Maclean, 2017; Fan et al., 2022). In the lung and systemic circulation, hvKp likely needed aerobactin and yersiniabactin to thrive against host defenses (Russo and Marr, 2019) (neutrophils, transferrin/lactoferrin sequestering iron, etc.), and indeed the patient’s initial infection was severe even with those factors. By the time the eye was infected, the ecological context had shifted: the injured, immune-privileged eye offers nutrients (the vitreous humor contains utilizable substrates and relative anoxia (Shui et al., 2009) tolerated by *K. pneumoniae*) and comparatively lower immune surveillance (Streilein, 2003). By contrast, the heavy-metal resistance (HMR) determinants detected in the ocular cKp isolate comprise compact modules (predominantly chromosomal or in small accessory cassettes) and therefore entail a much smaller biosynthetic cost than a large virulence plasmid. In this damaged niche—where inflammatory cells, tissue breakdown, and residual antimicrobials can generate oxidative stress and copper/zinc fluxes—HMR systems (e.g., Cu/Zn efflux and detoxification regulons) likely improve persistence by increasing tolerance to host-imposed metal intoxication and environmental stress (Djoko et al., 2015), yielding a net fitness benefit for cKp. Consistent with a payload-dependent cost, laboratory studies have shown that virulence plasmids can incur a fitness: for example, a study on a hybrid virulence plasmid observed it could not be stably maintained over serial passages without selection, due to the burden on the bacterium (Fan et al., 2022). The same study also noted that adding conjugation machinery to a virulence plasmid (thus increasing its size) affected stability (Fan et al., 2022).

### 4.4 Plasmid ecology and the risk of virulence–resistance convergence

Our genomic comparisons also shed light on the plasmid ecology of these strains. Consistent with prior studies, the hvKp strain’s plasmid (pLung) is non-conjugative (Gu et al., 2018; Tian et al., 2021), which likely limits its spread to other strains (and indeed the ST133 strain did not acquire it). On the other hand, the ST133 strain’s plasmid (pEye) appears to be a more mobile element (IncFIBK/FIIK) commonly found in multidrug-resistant *K. pneumoniae* lineages (Nguyen et al., 2024). The finding that ST133 retained a conjugative plasmid (albeit with partial tra genes) raises the concern that, in different circumstances, classical strains could acquire virulence genes or vice versa. Recent reports have warned of convergence between hvKp and classical MDR strains via plasmid exchange (Nguyen et al., 2024). Fortunately, in this patient’s case, the two strains did not swap genetic material. Had the virulence plasmid been transferable, the ST133 strain might have become a highly virulent hybrid. This scenario underscores a broader public health point: the co-existence of multiple *K. pneumoniae* strains in one host provides an opportunity for horizontal gene transfer (Shankar et al., 2022). Vigilance is needed to monitor for the emergence of such hybrid strains that combine hypervirulence and drug resistance (Russo and Marr, 2019).

### 4.5 Clinical implications and surveillance recommendations

This case highlights a few important points for clinicians and microbiologists.

#### 4.5.1 Early identification of hvKp—clinical first

hvKp syndrome should be suspected on clinical grounds and followed by a prompt, systematic search for multi-organ involvement. Practical triggers include community-onset invasive infection with rapid deterioration (Choby et al., 2020); cryptogenic liver abscess (Siu et al., 2012) or pneumonia with signs of metastatic seeding (e.g., eye or CNS); severe sepsis in patients with diabetes or other classic risk factors (Siu et al., 2012); and discordant treatment responses between localized foci and systemic disease (Russo and Marr, 2019). When these features are present, clinicians should not wait for laboratory confirmation: initiate targeted molecular testing in parallel with early cross-sectional imaging, urgent ophthalmologic assessment when indicated, and appropriate source control. In our case, early detection of *rmpA2* and *iutA* supported risk stratification and aggressive therapy; however, the eye—relatively sheltered from systemic antibiotics and immune responses—progressed to an irreparable stage, underscoring the need for timely focal intervention. (e.g., intravitreal antibiotics or surgical drainage).

#### 4.5.2 Using virulence markers appropriately — adjuncts, not arbiters

PCR/rapid tests for *rmpA/rmpA2, iucA* (and related markers) are valuable adjuncts to flag hvKp, but single-site, single-timepoint results may miss hvKp or invasive cKp. Within-host change (e.g., plasmid loss/gain) and sampling site effects can yield marker discordance across serial specimens. When clinical suspicion is high yet markers are negative—or when earlier positives turn negative—further investigation is warranted rather than assuming laboratory error. We recommend repeat testing and, where feasible, molecular assessment of multiple compartments (e.g., blood and metastatic foci) to capture gene context. Definitive classification should integrate clinical phenotype, imaging, source control findings, and molecular data rather than any single test.

#### 4.5.3 Monitoring plasmid evolution

From both research and public-health perspectives, prompt detection of plasmid loss or acquisition in hvKp is pivotal to preventing the emergence of hypervirulent and multidrug-resistant strains. Although hvKp plasmids are often regarded as relatively stable in the community (Russo and Marr, 2019), exposure to diverse environments (e.g., hospitals, antibiotic selection, distinct host niches) can drive within-host and within-ward evolution (Zhou et al., 2023). Conceptually, loss of a virulence plasmid may attenuate virulence (Zhou et al., 2023) yet open ecological space for resistance plasmid acquisition, while classical clones can conversely gain virulence plasmids and become hvKp—both trajectories have been described in *K. pneumoniae* (Gu et al., 2018). Capturing such gain/loss events in real time informs patient management and surveillance. Many outbreak investigations still rely solely on short-read NGS, which often fragments plasmids and obscures the chromosomal/plasmid context of key virulence and resistance genes. By contrast, long-read platforms or hybrid assemblies can deliver strain-level, circularized (gap-free) plasmid reconstructions with same-day turnaround in routine settings, and are therefore increasingly advantageous for plasmid monitoring. When adequate read accuracy and depth are achieved, long-read or hybrid WGS should be prioritized for: (i) suspected hvKp with rapid clinical deterioration; (ii) within-host phenotype switches (e.g., virulence-marker discordance across serial samples) where plasmid dynamics may explain the discrepancy; and (iii) clusters/outbreaks in which plasmid-borne traits are epidemiologically relevant. These practices support active surveillance, clarify evolutionary pathways during infection, and improve the actionability of genomic results for both clinicians and infection-control teams.

#### 4.5.4 Long-read accuracy and scope

Historically, long-read platforms have lower per-read accuracy with homopolymer indels, which can affect SNP-level tasks if single reads are interpreted directly (Delahaye and Nicolas, 2021). In our workflow, typing (MLST) and plasmid reconstruction relied on depth-polished consensus (not single reads), consistent with recent reports that nanopore-only pipelines can support surveillance-grade genotyping in Klebsiella under modern QC (Foster-Nyarko et al., 2023; Sanderson et al., 2024). For SNP-level outbreak resolution, hybrid short+long-read sequencing or validated high-accuracy long-read pipelines (e.g., duplex/HiFi) are preferable (Shafin et al., 2021; Wenger et al., 2019). We have therefore framed long-read data as consensus-driven for typing and context, and avoided over-interpretation at single-SNP resolution (Foster-Nyarko et al., 2023).

## Data Availability

The datasets presented in this study can be found in online repositories. The names of the repository/repositories and accession number(s) can be found in the article/**Supplementary Material**.
